# Effect of carbohydrate substrates on growth and enterotoxin gene expression in *Bacillus cereus* (*pacificus*)

**DOI:** 10.1038/s41598-025-31689-5

**Published:** 2025-12-10

**Authors:** Katerina Vyklicka, Jiri Kucera, Vojtech Barton, Jan Bohm, Roman Reminek, Petra Kubasova, Katerina Paskova, Zdenek Glatz, Jan Lochman, Filip Ruzicka, Petra Borilova Linhartova

**Affiliations:** 1 RECETOX, Faculty of Science, Masaryk University, Kotlarska 2, Brno, Czech Republic; 2 Department of Biochemistry, Faculty of Science, Masaryk University, Kotlarska 2, Brno, Czech Republic; 3 Institute of Analytical Chemistry of the Czech Academy of Sciences, v. v. i., Veveri 97, Brno, Czech Republic; 4 Institute of Microbiology, St. Anne’s University Hospital in Brno, Pekarska 53, Brno, Czech Republic

**Keywords:** Gastrointestinal illness, Bacterial toxin, Lactose, Transcriptome, RNA-seq, Storage, Human milk, Biotechnology, Microbiology, Molecular biology

## Abstract

*Bacillus cereus sensu lato* is a common contaminant of improperly stored food and human milk, capable of causing severe emetic and diarrheal diseases, particularly in infants and immunocompromised individuals. In this in vitro study, the effects of various temperatures and carbohydrate substrates on the growth of *B. cereus* (*pacificus*) strain ATCC 10987 and its expression of enterotoxin genes were explored. Bacterial growth at several temperatures and with six different carbohydrate substrates was evaluated. In cultures grown with various substrates at 37 °C, selected metabolites were analyzed using capillary electrophoresis and RNA-seq transcriptomic profiling was performed. At the same temperature, glucose, fructose, sucrose, and xylitol-enriched environments were preferable for the bacterial growth to lactose and galactose-enriched media. Lactate production markedly increased in bacterial cultures grown with glucose, fructose, or sucrose, accompanied by a drop in pH and increased substrate consumption. The lowest expression of non-hemolytic enterotoxin genes (*nhe*) was observed in glucose and lactose-enriched media, while downregulation of enterotoxin FM gene (*entFM*) expression was found in bacteria cultured with lactose. Other known enterotoxin genes, such as hemolysin BL (*hbl*) and cytotoxin K (*cytK*), were not expressed by this bacterial strain under any of the tested conditions. In conclusion, a lactose-rich environment slowed the growth of *B. cereus* (*pacificus*) ATCC 10987 and was associated with a downward trend in the expression of the enterotoxin genes *nhe* and *entFM*. These findings indicate that the presence of lactose, a dominant carbohydrate of human milk, may influence the pathogenic potential of *B. cereus s. l.*

**Supplementary Information:**

The online version contains supplementary material available at 10.1038/s41598-025-31689-5.

## Introduction

*Bacillus cereus sensu lato* (*s. l.*) is a Gram-positive, endospore-forming, facultatively aerobic bacterium^1^, commonly found in soil and on growing plants^2^. It has been isolated from a wide variety of foods and food ingredients, such as rice, meat, spices, dried foods, vegetables, dairy products, and milk (including human milk)^3–7^, particularly when they are stored improperly^8^.

*B. cereus* is known for producing a range of virulence factors, including several enterotoxins. Its two most common enterotoxins, Nhe (non-hemolytic enterotoxin) and Hbl (hemolysin BL), are known to cause foodborne illnesses. Although the gastrointestinal symptoms caused by these toxins are usually mild and self-limiting^2^, the risks should not be underestimated, especially in vulnerable individuals such as infants^4,9–11^. Several fatal cases have been reported^12,13^. Recently, *B. cereus s. l.* was recently identified as the leading cause (19%) of food poisoning outbreaks^14^.

The remarkable environmental resilience of *B. cereus s. l.* is one of the reasons it poses such a challenge. Due to its spore-forming ability, it can withstand a wide range of environmental stress conditions^15^, including low pH^16^, and high temperatures. It remains viable even after boiling^17^ and is capable of growing over a wide temperature range from 4 °C to 50 °C, with optimal growth observed between 30 °C and 40 °C^18,19^. It can also adapt to the mammalian gastrointestinal environment^7^.

In addition to temperature, the type of available carbon source significantly affects both bacterial growth and expression of its genes including those encoding the production of bacteriocins^20^. Previous studies have shown that enterotoxin production in *B. cereus s. l.* is influenced by nutrient availability^21,22^. Thus, a deeper understanding of how metabolic conditions modulate toxin expression is essential. However, the complexity of regulatory networks and potential interactions with other virulence factors makes it difficult to predict toxicoinfection risk based solely on genomic data^23^.

In this context, we hypothesized that carbohydrate sources influence both the growth of *B. cereus s. l.* and the production of its enterotoxins. This in vitro study was designed to: (i) conduct a comparative experiment to identify conditions, specifically temperature and carbohydrate type, that promote the growth of a *B. cereus* strain; and (ii) investigate the expression of genes encoding enterotoxins in response to the substrate availability under simulated in vivo conditions.

## Methods

The following analyses were performed in this study: (i) confirmation of bacterial strain identity using whole genome sequencing (WGS); (ii) assessment of bacterial growth dynamics on various carbohydrates at different temperatures; (iii) analysis of carbohydrate consumption and organic acids (OA) production by capillary electrophoresis; and (iv) transcriptomic profiling using the RNA-seq technique, with a particular focus on enterotoxin genes expression.

Six mono- or disaccharides common in human diet were selected for this study. Glucose, fructose, galactose, lactose, and sucrose represent the most common monosaccharides and disaccharides in the human diet^24^. They are also the main naturally occurring carbohydrates with well-established metabolic pathways in humans. In addition, xylitol was included as a representative of non-caloric sweeteners commonly used in foods and pharmaceutical products^25^.

### Bacterial strain and culture conditions

Based on our recent study on human milk storage^8^, *B. cereus s. l.*, a common contaminant of stored human milk, was selected for a growth experiment, metabolite analysis, and transcriptomic analysis. The *B. cereus* ATCC 10987 strain was cultured on Columbia agar with 5% sheep blood (catalog No. KA5, LabMediaServis, CZ) at 37 °C overnight. A single colony from the blood agar plate was inoculated into a 15-mL TubeSpin bioreactor tube (TPP) containing 5 mL of Brain Heart Infusion (BHI) broth (Merck, DE). The culture was incubated overnight at 37 °C with agitation at 200 rpm to establish the stock bacterial culture, which was subsequently cryopreserved in 25% glycerol at − 80 °C for long-term storage.

### Whole genome sequencing-based characterization of the bacterial strain

Genomic DNA was isolated from the stock culture obtained from a single colony of *B. cereus s. l.* grown on blood agar. Detailed information on DNA extraction, sequencing, and genome analysis is provided in Supplement 1 (Methods) and Supplement 2 (Table S1).

### Growth kinetics of *B. cereus* culture with various carbohydrates

To investigate the influence of different carbohydrates on the growth of *B. cereus s. l.*, a low-carbohydrate culture medium, “Tryptic Soy Broth without Dextrose” (TSB–w/oD, Sigma-Aldrich, USA), was used for its versatility and suitability for controlled carbohydrate supplementation. Compared to Brain Heart Infusion (BHI), TSB–w/oD medium offered a more defined background for examining the effects of added carbohydrates. A stock culture of *B. cereus s. l.* was first pre-cultured overnight at 37 °C in a 50 mL polypropylene tube (TPP) containing 35 mL of TSB–w/oD (the maximum recommended working volume) under constant agitation at 200 rpm. The resulting stationary-phase culture was washed once with fresh TSB–w/oD and then used as an inoculum (initial OD_600_ adjusted to 0.1). The bacterium was then cultured in 35 mL of TSB–w/oD supplemented with selected carbohydrates at 0.5% (w/v), including fructose, galactose, glucose, lactose, sucrose, and xylitol (Merck, DE). Incubations were carried out in biological triplicates at different temperatures in a refrigerated incubator equipped with a platform shaker (200 rpm) to simulate variable storage conditions. At one-hour intervals, 0.2 mL samples were collected, and OD_600_ was measured in 96-well plates using a microplate reader ELX808 (BioTek Instruments, USA) in triplicates from each biological replicate. All experiments were performed under aerobic conditions in ambient air and conducted in biological triplicates, ensuring statistical robustness and relevance to real-world food storage environments. To model the growth kinetics of *B. cereus s. l.*, the modified Gompertz growth model was applied to the OD₆₀₀ experimental data. Curve fitting was performed using nonlinear regression in GraphPad Prism (version 10.4.1, GraphPad Software) to estimate key growth parameters, including the maximum specific growth rate (µ_max_), *lag* phase duration (λ), bacterial doubling time (T_d_), and the maximum population density (Y_max_), expressed as the highest measured optical density at 600 nm (OD_600_). The quality of the model fit was evaluated using standard statistical metrics, including degrees of freedom (DF), coefficient of determination (R^2^), residual sum of squares (SS), and standard error of the estimate (Sy.x).

### RNA-seq-based transcriptomic profiling

#### Culture of *B. cereus s. l.* for transcriptomic analysis

A stock culture of *B. cereus s. l.* was pre-cultured overnight at 37 °C in a 50-mL TPP containing 35 mL of TSB–w/oD, with agitation at 200 rpm. The resulting stationary-phase culture was washed once with fresh TSB–w/oD to remove residual nutrients and metabolic byproducts. This culture was then used as an inoculum for the main experiment. The starting optical density (OD₆₀₀) was adjusted to 0.01, and cells were inoculated into 35 mL of TSB–w/oD supplemented with 0.5% (w/v) carbohydrate, using one of the following carbon sources: fructose, galactose, glucose, lactose, sucrose, or xylitol. All culture experiments were performed in biological triplicates under aerobic conditions at 37 °C in a 5% CO_2_-enriched atmosphere, using CelCulture CO_2_ incubator (Esco Lifesciences, USA) with continuous shaking at 200 rpm. This setup was used to simulate in vivo physiological conditions of the human body. After 12 h of incubation, the biomass was harvested by centrifugation at 8,000 × *g* for 10 min at 4 °C and immediately processed.

#### Total RNA isolation, library preparation, and sequencing

Total RNA was isolated using the TRI reagent (Merck, DE) and treated with the RapidOut DNA Removal Kit (ThermoFisher Scientific, USA) to eliminate contaminating DNA. RNA quality and quantity were assessed using a Qubit 4 fluorometer with the Qubit RNA HS and RNA IQ Assay Kits (ThermoFisher Scientific, USA) and a Fragment Analyzer with the HS RNA Kit (Agilent Technologies, USA).

cDNA libraries were constructed using the ScriptSeq Complete Kit for Bacteria (Illumina, USA), which includes Ribo-Zero technology for ribosomal RNA removal and the ScriptSeq v2 RNA-Seq Library Preparation Kit. The quality and quantity of the cDNA libraries were assessed using a Qubit 4 fluorometer with the Qubit DNA HS Assay Kit (ThermoFisher Scientific, USA) and a Fragment Analyzer with the HS DNA Kit (Agilent Technologies, USA). Two samples (one from the glucose culture and one from the xylitol culture) did not pass quality control and were, therefore, excluded from sequencing. The remaining cDNA libraries were diluted to a final concentration of 10 nM. Sequencing was performed using the NextSeq 500/550 High Output Kit v2.5 (150 cycles; Illumina, USA), which generated 75 bp paired-end reads.

#### Bioinformatic analysis

The data was processed using nf-core/rnaseq v3.14.0 from the nf-core collection of workflows^26^, utilizing reproducible software environments provided by the Bioconda^27^ and Biocontainers^28^ projects. FeatureCounts^29^ was used to generate a feature count matrix across all growth conditions. The resulting data was further analyzed using nf-core/differential-abundance v1.4.0^26^. The DESeq2 module output was used for Gene Set Enrichment Analysis (GSEA) using fgsea, with gene set annotations obtained from KEGG^30^. KEGG pathway gene sets for *B. pacificus* were obtained via the EnrichmentBrowser R package (getGenesets(“bpac”, “kegg”)). KEGG data were sourced from the Kyoto Encyclopedia of Genes and Genomes database^30^, and gene set retrieval was performed using EnrichmentBrowser^31^.

#### Biostatistical analysis

Differential gene expression analysis (i.e., expression in bacteria grown on substrates compared to TSB without added carbohydrates serving as a baseline) was performed using the DESeq2 package in R. To control for false discoveries, *p*-values were adjusted using the Benjamini-Hochberg correction, and genes with an adjusted *p*-value (*p*_adj_) < 0.05 were considered significantly differentially expressed.

### Metabolite analysis by capillary electrophoresis

Samples for the determination of selected metabolites were collected at the beginning and after 12 h of *B. cereus* *s. l. *culture with different carbohydrates and frozen at − 80 °C. In addition, pH values were measured using a semimicro pH electrode (ThermoFisher Scientific, USA) and pHTestr 10BNC (Eutech Instruments, Singapore). The concentrations of six carbohydrates (fructose, galactose, glucose, lactose, sucrose, and xylitol) and five OA (formate, acetate, propionate, lactate, and butyrate) were determined by capillary electrophoresis (CE) with indirect detection using a method adopted from^20,32^. Detailed information on sample preparation and the final CE analysis conditions is provided in Supplement 1 (Methods).

## Results

### Identification of the *B. cereus* strain

The genome of the *B. cereus s. l.* strain used in our study was successfully sequenced using WGS and identified as *Bacillus cereus* ATCC 10987. However, it is important to note that during the preparation of this manuscript, the strain *B. cereus* ATCC 10987, characterized by unique metabolic features, such as urease production and xylose utilization, which distinguish it from other *B. cereus* strains, was removed from the RefSeq database following its reclassification as *Bacillus pacificus.* However, due to the lack of supporting literature on *B. pacificus* and the continued recognition of this strain as *B. cereus* ATCC 10987 within the scientific community, we refer to the strain as *B. cereus s. l.* throughout this study for clarity and consistency.

### Growth of *B. cereus* ATCC 10987 strain on various carbohydrates and at different temperatures

**Fig. 1:**
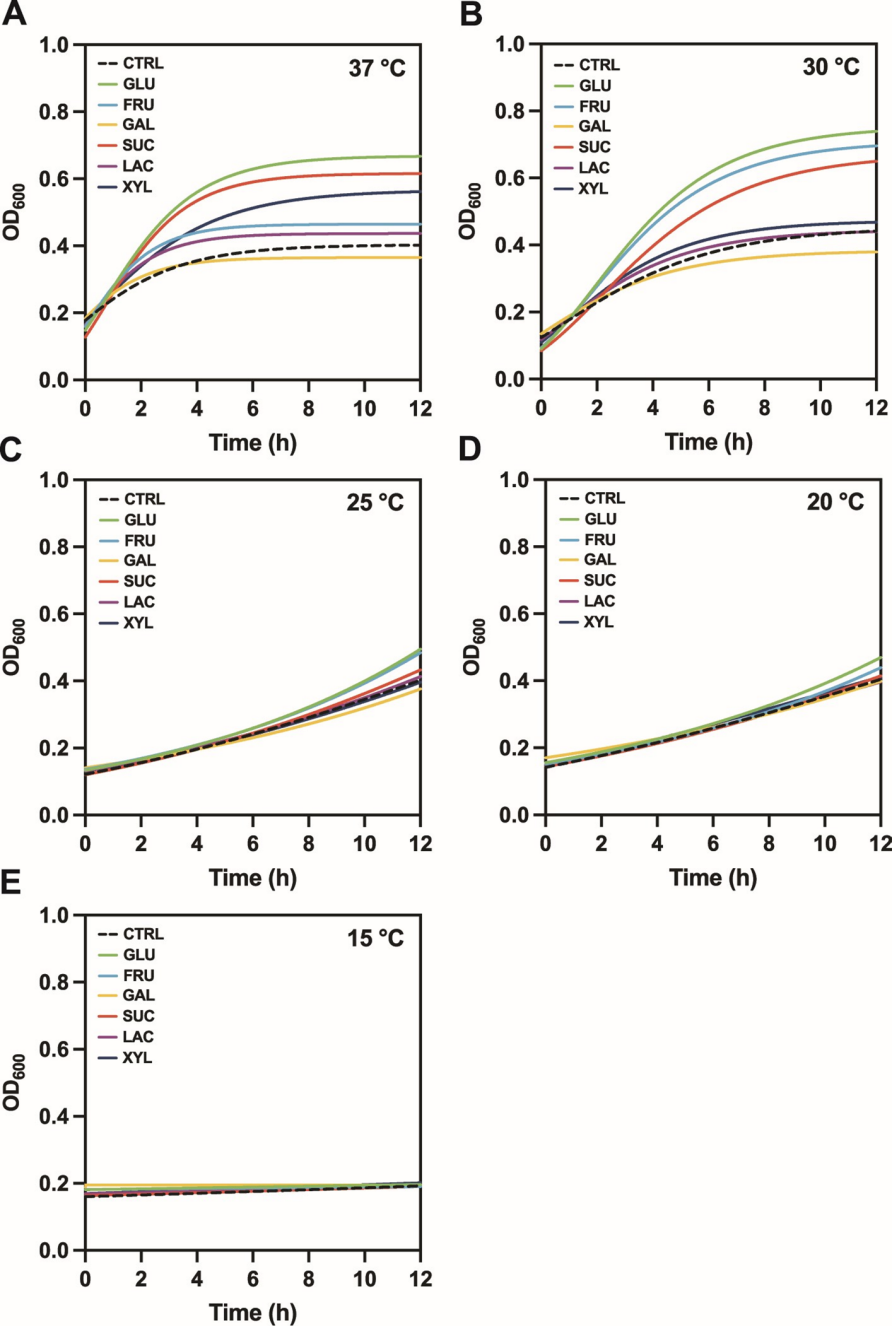
Growth curves of *Bacillus cereus* ATCC 10987 in the presence of various carbohydrates at different incubation temperatures. *B. cereus* was cultured in control medium supplemented with individual carbohydrates at 37 °C (**A**), 30 °C (**B**), 25 °C (**C**), 20 °C (**D**), and 15 °C (**E**). Carbohydrates: fructose (FRU, light blue), galactose (GAL, yellow), glucose (GLU, green), lactose (LAC, purple), sucrose (SUC, red), xylitol (XYL, dark blue), and control with no carbohydrate added (CTRL, dashed black line). The modified Gompertz model was applied to the experimental data. Statistical evaluation of the model fitting is provided in Supplement 2 (Table S3).

Growth curves of *B. cereus* ATCC 10987 were generated using six carbohydrate substrates, alongside a low-carbohydrate control medium TSB–w/oD, under a range of culture temperatures (Fig. 1). The resulting kinetic parameters are summarized in Supplement 2 (Table S2), and the statistical evaluation of the Gompertz model fit is presented in Supplement 2 (Table S3). At the optimal temperature of 37 °C, the highest specific growth rates (µ_max_) were observed in the following order: sucrose (0.14 h^− 1^), glucose (0.13 h^− 1^), fructose (0.13 h^− 1^), lactose (0.11 h^− 1^), galactose (0.09 h^− 1^), and xylitol (0.08 h^− 1^). The observed trend in µ_max_ was inversely proportional to T_d_. The maximum optical densities (Y_max_) at 37 °C ranged from 0.67 to 0.37 (in the order glucose, sucrose, xylitol, fructose, lactose, and galactose). Decreasing incubation temperature was associated with a consistent decline in µ_max_ accompanied by extended lag phases (λ) and longer doubling times (T_d_) across all carbohydrate substrates.

### Carbohydrate utilization by *B. cereus* ATCC 10987 strain at optimal temperature conditions

*B. cereus* ATCC 10987 strain exhibited the highest metabolic activity when cultured with glucose, fructose, and sucrose as carbon sources. These conditions led to the greatest OA accumulation, particularly lactate, acetate, and formate (Table 1). In contrast, butyrate and propionate remained below the limits of quantification (LOQ) or detection (LOD), as determined by stepwise dilution of analyte concentrations. The elevated production of acids, indicative of fermentative metabolism, corresponded with a significant decline in culture pH (Table 2). Carbohydrate consumption was observed in all tested media, with glucose showing the highest uptake (49.60%) and galactose the lowest (3.45%) (Table 1). Statistical evaluation of the results obtained is discussed in Supplement 1.

**Table 1:** Organic acids (OA) production and carbohydrate consumption by *Bacillus cereus* ATCC 10987 strain grown at 37 °C with various carbohydrates.

Carbohydrate	OA^a^ [mM] at t = 12 h					Carbohydrate^b^ [g L^− 1^]		Consumption
	Formate	Acetate	Propionate	Lactate	Butyrate	t = 0 h	t = 12 h	[%]
Fructose	2.37 ± 0.12	15.30 ± 0.29	< LOD	15.20 ± 0.41	< LOD	4.94 ± 0.10	3.32 ± 0.16	32.80
Galactose	2.48 ± 0.33	12.10 ± 1.15	< LOD	6.41 ± 1.06	< LOD	5.02 ± 0.12	4.85 ± 0.11	3.45
Glucose	1.26 ± 0.13	11.60 ± 0.43	< LOD	18.40 ± 0.48	< LOQ	4.95 ± 0.17	2.49 ± 0.10	49.60
Lactose	2.74 ± 0.13	10.40 ± 0.76	< LOD	4.99 ± 0.25	< LOD	5.07 ± 0.16	4.74 ± 0.19	6.47
Sucrose	1.61 ± 0.06	11.50 ± 0.27	< LOD	13.20 ± 0.72	< LOD	4.97 ± 0.13	3.10 ± 0.19	37.70
Xylitol	2.62 ± 0.24	8.02 ± 0.46	< LOD	5.67 ± 0.45	< LOD	5.10 ± 0.16	4.85 ± 0.21	4.95

Note: The data are presented as means of eight results (*n* = 8), derived from four independent samples that were each analyzed in duplicate. Abbreviations: t, incubation time; LOQ, limit of quantification; LOD, limit of detection. ^a^Mean of 8 independent analyses of the culture media ± standard deviation (SD). ^b^Mean of 6 independent analyses of the culture media ± SD.

**Table 2:** pH changes during the culture of *Bacillus cereus* ATCC 10987 strain with various carbohydrates.

Carbohydrate	pH_0_	pH_12_
Fructose	7.12 ± 0.03	5.63 ± 0.03
Galactose	7.10 ± 0.01	6.55 ± 0.01
Glucose	7.12 ± 0.01	5.37 ± 0.02
Lactose	7.19 ± 0.02	6.45 ± 0.03
Sucrose	7.15 ± 0.01	5.56 ± 0.03
Xylitol	7.18 ± 0.01	6.56 ± 0.01

Note: The experiments were performed in quadruplicates at 37 °C. All values are presented as mean ± standard deviation (*n* = 3). *pH*_*0*_, initial pH before incubation; pH_12_, final pH after 12-hour incubation.

### Global transcriptomic response of *B. cereus* ATCC 10987 strain to carbohydrate sources

To better understand the molecular mechanisms underlying substrate-dependent growth patterns observed at 37 °C (section “Growth of *B. cereus* ATCC 10987 strain on various carbohydrates and at different temperatures”), gene expression profiling and targeted metabolite analysis were performed on cultures of *B. cereus* ATCC 10987 strain grown with individual carbohydrate substrates.

**Fig. 2:**
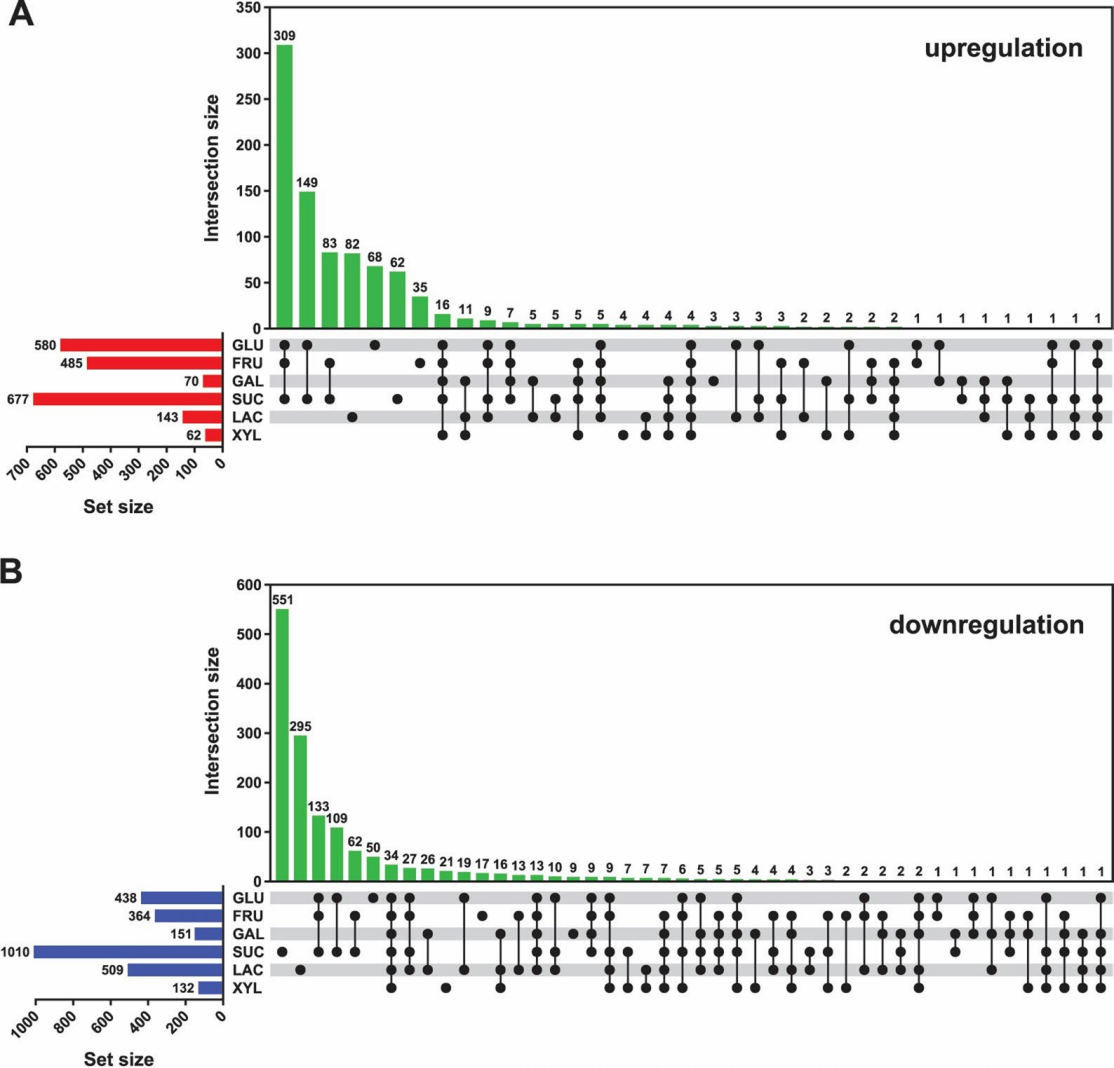
Transcriptomic analysis of *Bacillus cereus* ATCC 10987 grown on various carbohydrates at 37 °C. UpSet plots showing the number and overlap of significantly upregulated (**A**) and downregulated (**B**) genes across the carbon sources tested. Note that sucrose, glucose, and fructose affected a relatively large set of genes, showing broadly similar transcriptional responses. Besides, sucrose uniquely repressed a group of genes not downregulated by any other tested carbohydrate; to a lesser extent, lactose exhibited the same trend.

A total of 5,010 genes were identified through transcriptomic analysis (Supplement 2 – Table S4). The significantly differentially expressed genes (DEGs) of studied bacterial strain under each condition are summarized in Supplement 2 (Table S5). The analysis of gene regulation patterns revealed considerable overlap among cultures grown in glucose, fructose, and sucrose (Fig. 2) both where the number of upregulated and downregulated genes is concerned. Cultures in sucrose showed the highest number of upregulated as well as downregulated genes, indicating a high impact of this sugar on the metabolism of *B. cereus*. Lactose substrate also showed a high number of genes that were downregulated by this but not by any other carbohydrate.

**Fig. 3:**
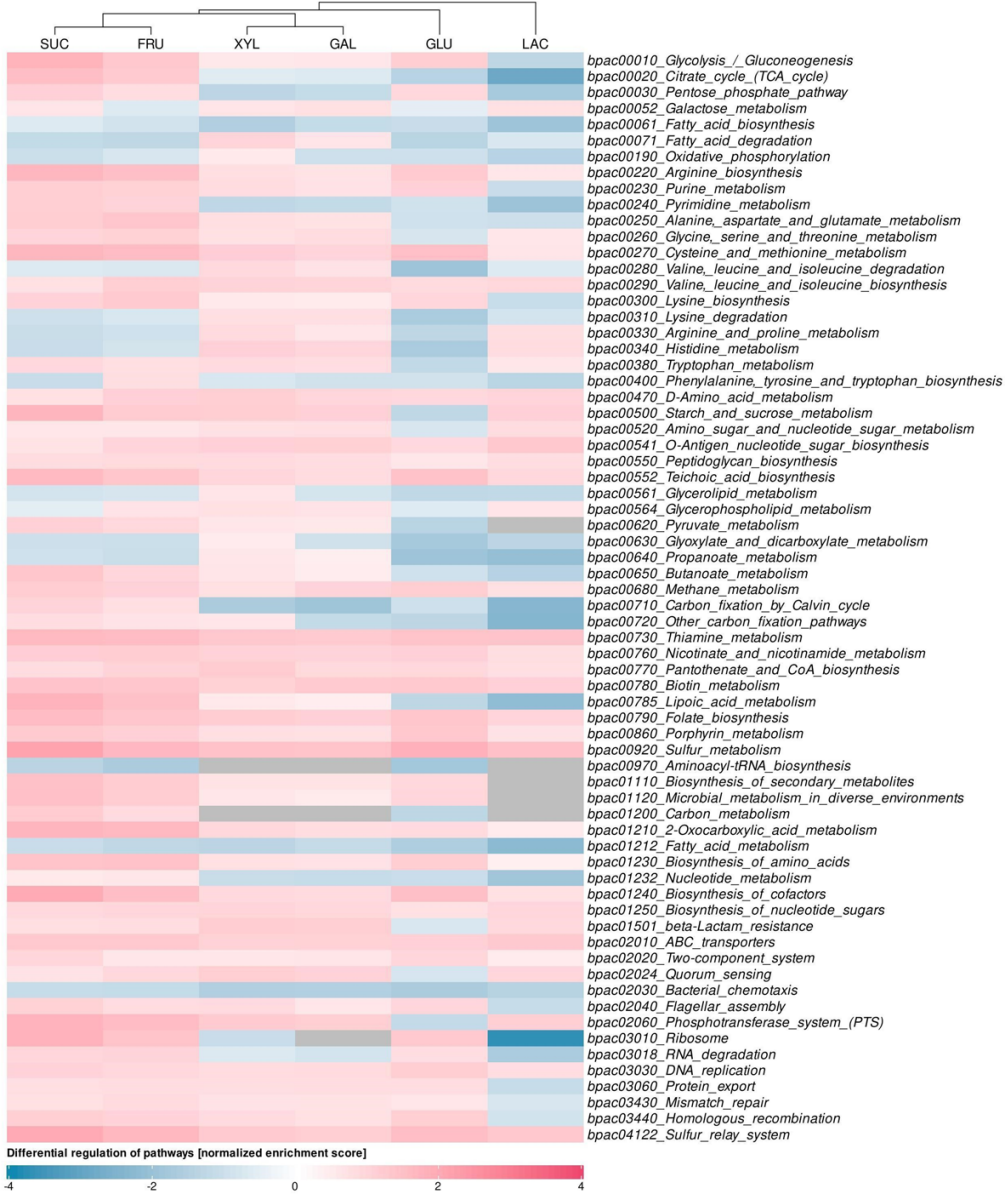
Metabolic pathways differentially expressed in *Bacillus cereus* ATCC 10987 grown on various carbohydrates at 37 °C. The colour scale indicates the extent of up/downregulation, grey indicates that the enrichment score could not be calculated. Note the results of the clustering analysis above the heatmap.

Figure 3 presents several clusters of metabolic pathways that were differentially regulated in response to individual carbohydrates. One major cluster, comprising sucrose and fructose, was characterized by the upregulation of the citrate cycle (TCA cycle), pyrimidine metabolism, and carbon fixation via the Calvin cycle. Glucose showed a partial overlap with this cluster, especially in the upregulation of the pentose phosphate pathway, certain amino acid biosynthesis pathways, and RNA degradation. In parallel, glucose cultures exhibited increased expression of glycolytic genes and comparatively lower expression of genes involved in the TCA cycle and oxidative phosphorylation than cultures grown on other sugars. This relative pattern aligns with overflow metabolism and CcpA-mediated carbon catabolite repression under high glycolytic flux^33^. Potential oxygen-transfer limitation in batch culture may also have contributed, although dissolved oxygen was not monitored^34^.

The second cluster consisting of xylitol and galactose, showed increased expression of genes involved in propanoate (propionate) metabolism, fatty acid degradation, and lysine degradation. Interestingly, lactose-grown cultures exhibited a partially similar pattern, and all three carbohydrates (lactose, xylitol, and galactose) exhibited the slowest growth of *B. cereus* ATCC 10987 strain. These three carbohydrates shared the downregulation of the pentose phosphate pathway and RNA degradation, and upregulation of selected amino acid biosynthesis pathways.

Lactose and glucose did not clearly belong to either cluster, although they shared specific regulatory patterns, most notably the downregulation of genes involved in propionate, butanoate (butyrate), lipoic acid, and alanine, aspartate, and glutamate metabolism. The most significantly differentially expressed genes from these pathways are shown in Supplement 1 (Figure S1).

#### Expression of *B. cereus* ATCC 10987 strain toxins in response to carbohydrate sources

**Fig. 4:**
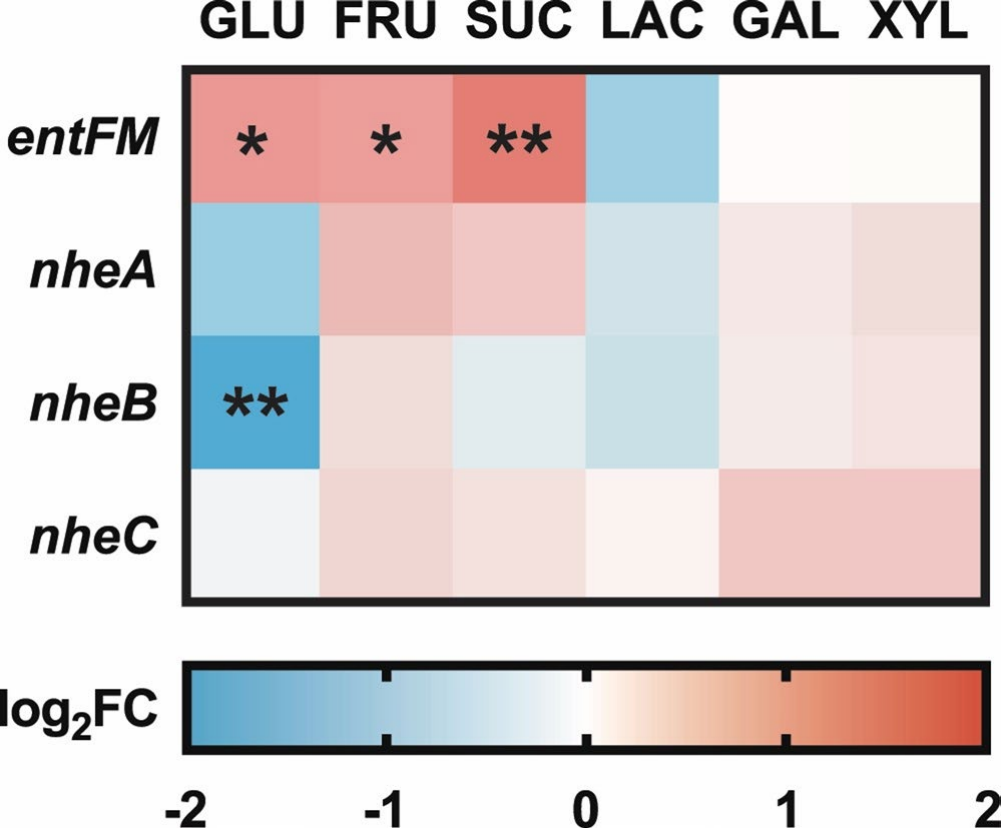
Expression of enterotoxin-encoding genes in *Bacillus cereus* ATCC 10987 grown on various carbohydrates at 37 °C. Statistical significance is indicated as follows: *p*_adj_ ≤ 0.05 (*), *p*_adj_ ≤ 0.01 (**).

Transcriptomic analysis revealed the presence of gene transcripts encoding several enterotoxins, including Nhe, EntFM, pre-toxin TG, and the LXG polymorphic toxin, while no expression of *cytK* and *hbI* genes was detected. The *pre-toxin TG* and *LXG polymorphic toxin* genes remained consistently low and unaltered across all tested substrates (data not shown). On the other hand, the expression of *nhe* and *entFM* genes varied across different carbohydrate conditions; however, only a few of these changes met the threshold for significantly differential expression. The greatest downregulation of genes *nheA*, *nheB*, and *nheC* genes was detected in the bacteria grown in glucose-supplemented substrate, followed by lactose. The lowest expression of the *entFM* gene was observed in lactose, followed by galactose and xylitol substrates, while the highest was detected in sucrose, glucose, and fructose (see Fig. 4). These results suggest that the expression of toxins of this bacterial strain is modulated by carbohydrate availability, with glucose and lactose potentially suppressing the production of key enterotoxins.

## Discussion

*B. cereus s.l.* is a well-recognized foodborne pathogen capable of causing emetic and diarrheal diseases^35^. Its pathogenic potential is linked to its remarkable adaptability to a wide range of environmental conditions, including pH, temperature, and available growth substrates, allowing *B. cereus* to persist in diverse food systems and environments^16,36^.

The strain investigated in this study, originally classified as *B. cereus* ATCC 10987 (Group III), has recently been reclassified as *B. pacificus*, yet we refer to it here as *B. cereus s. l.* in alignment with its established identity in prior food microbiology literature^37^. Known for its strong spore resistance and broad growth temperature range (15–45 °C), this strain has been linked to foodborne pathogenicity, including incidents in dairy and human milk storage environments^36^(Pivrncova et al., 2025).

Our growth experiments confirmed that *B. cereus* ATCC 10987 strain was capable of growing within a temperature range of 15–37 °C, although growth was suboptimal at lower temperatures. Previous studies have reported a broader growth range, from as low as 4 °C^38^ to as high as 46 °C^18,19^, with optimal growth typically observed between 30 °C and 40 °C^35^.

In our study, the most rapid onset of growth occurred at 37 °C, although the highest Y_max_ values were observed at 30 °C. At 15 °C, growth was detectable but significantly slower, characterized by an extended *lag* phase. The ability of several potentially toxigenic strains to grow at temperatures below 10 °C has also been previously documented^18^. However, unlike in the study by^39^, who found that low temperature had little effect on the final biomass yield, our cultures incubated at 15 °C showed significantly lower optical densities than those observed at 25 °C, 30 °C, or 37 °C during the 12-hour incubation with various carbohydrates. This suggests that while *B. cereus s. l.* tolerates low temperatures, prolonged incubation or stress may inhibit full metabolic activation.

In general, *B. cereus s. l.* can adapt to a wide range of growth conditions, including anaerobic environments and various energy sources available in the gastrointestinal tract^36^. Under anaerobic conditions, *B. cereus s. l.* is capable of fermenting carbohydrates such as glucose, sucrose, and fructose, as well as certain amino acids^36^. Our growth data indicate a clear preference for glucose, sucrose, and fructose over xylitol, lactose, and galactose. Although *B. cereus* ATCC 10987 strain was able to grow on all tested carbohydrates, the three fermentable substrates (glucose, sucrose, fructose) are among the most accessible carbohydrate energy sources in the human gastrointestinal tract^40^. Furthermore, Carlin et al.^36^ described the bacterial preference for fermentable carbohydrates, particularly under suboptimal growth conditions, which results in increased production of fermentation end products such as acetate, formate, propionate, and lactate^36^. This metabolic activity leading to the acidification of the environment, which may contribute to spoilage in susceptible products. In contrast, galactose and xylitol supported the slowest growth and minimal OA production, indicating inefficient metabolic processing of these substrates^41^. As expected, the highest levels of fermentation products were measured in cultures supplemented with glucose, sucrose, and fructose.

Warda et al. found that *B. cereus* ATCC 10987 strain possessed the genomic potential for the utilization of glucose, fructose, and xylose, whereas it lacked the genetic basis for the growth on lactose^37^. However, unlike our findings, they reported that this strain was unable to grow in sucrose and galactose. This discrepancy may be explained by the fact that *B. cereus* is capable of growing in a low-carbohydrate, amino acid-rich, medium (such as the TSB used in our study) even in the absence of added carbohydrates^36^. Interestingly, we observed that the bacteria grew better in the control medium than in the same medium supplemented with lactose or galactose. While high concentrations of certain carbohydrates can induce metabolic stress and inhibit bacterial growth^42^, this mechanism does not apply to *B. cereus* at carbohydrate concentrations used in our study^43^.

Transcriptomic analysis further highlighted the substrate-dependent metabolic reprogramming of *B. cereus* ATCC 10987 strain. Sucrose and fructose promoted upregulation of TCA cycle genes, pyrimidine metabolism, and carbon fixation pathways, whereas glucose enhanced expression in the pentose phosphate pathway and biosynthetic processes. Consistent with growth kinetics, *B. cereus *exhibited the most rapid proliferation in glucose-supplemented cultures, accompanied by upregulation of glycolytic genes and reduced expression of the TCA cycle and oxidative phosphorylation pathways. This pattern reflects the metabolic flexibility of facultative anaerobes, which under glucose excess or limited oxygen favor fermentative glycolysis over respiration. Such overflow metabolism supports rapid ATP generation and NAD^+^ regeneration via lactate dehydrogenase and mixed-acid branches. After 12 h, OAs accumulated to 31.26 mM while glucose decreased by 2.46 g/L (≈ 13.65 mM), yielding ≈ 2.29 mol organic acid per mol glucose consumed. A carbon recovery of ~ 97% in measured OAs, with the remainder likely in CO₂, biomass, and neutral metabolites, further supports this interpretation^33,34^. These patterns underscore how *B. cereus s. l.* tailors its central metabolism to efficiently utilize available carbon sources^41^. In contrast, growth on xylitol, galactose, and lactose was associated with upregulation of propionate metabolism, fatty acid degradation, and lysine catabolism – pathways potentially indicative of metabolic stress or adaptation to suboptimal energy sources.

*B. cereus-*related foodborne disease is primarily caused by its enterotoxins. Unlike the *nhe* gene cluster, which is present in virtually all *B. cereus s. l.* strains and constitutes the dominant diarrheal toxin, the *hbl* and *cytK* genes are found in only about 50% of strains^7^. In our sequencing data, neither *hbl* nor *cytK* were detected, while *nheA*, *nheB*, and *nheC* were present. This observation is consistent with previous findings for the *B. cereus* ATCC 10987 strain, which lacks the *hbl* operon but is known to express *nhe* and to exhibit strong cytopathogenic activity^44^. It should also be noted that *B. cereus s. l.* can produce additional enterotoxins, such as the pre-toxin TG and the LXG polymorphic toxin. The expression of the corresponding genes was, however, negligible in our experiment. Consistent with the findings of^45^, who studied *B. cereus s. l.* strains isolated from donor human milk and hospital environments^45^, we also observed differential expression of the *entFM* gene, alongside the *nhe* gene.

Ouhib et al. suggested that optimal growth conditions, characterized by high energy availability, suppress toxin expression, whereas stress or nutrient limitation may enhance it reported that adverse growth conditions, such as culture in galactose and xylitol^40^. In our study, the lowest expressions of the *nhe* genes were observed in glucose-supplemented medium, which supported the most robust growth and, therefore, represented the most favorable condition. The *entFM* gene, by contrast, exhibited upregulation under favorable growth conditions (glucose, fructose, sucrose), suggesting its expression may be linked to cellular activity or metabolic flux rather than classical stress responses. It is, therefore, possible that *entFM* serves a different biological function under these conditions than classical enterotoxins. Additionally, *entFM*, *nheA*, and *nheB* genes showed downward trends in expression in lactose, despite the low growth in this substrate. This observation is consistent with the findings by Rowan and Anderson (1997), who reported that lactose as the sole carbon source supported growth of *B. cereus s. l.* but not its toxin production^22^. This may be related to the disaccharide nature of lactose, which is composed of glucose and galactose. The observed expression pattern of *nhe* genes suggests a regulatory influence of the glucose moiety. It is also worth noting that glucose and lactose showed similar patterns of gene downregulation in pathways related to propionate, butyrate, lipoic acid, and alanine, aspartate, and glutamate metabolism.

From a food safety and quality perspective, these findings suggest that the formulation of food products, particularly carbohydrate content, can influence not only the growth kinetics of *B. cereus s. l.* but also its potential for toxin production. Such insights are relevant to risk mitigation strategies in dairy, infant nutrition, and carbohydrate-rich formulations.

We acknowledge several limitations. While the medium used was low in carbohydrates, residual saccharides from enzymatic digests (e.g., casein hydrolysate) may have influenced baseline growth in control conditions. Additionally, longer incubation periods may be necessary to fully capture growth dynamics at suboptimal temperatures, where the *lag* phase may last as long as several days. Furthermore, although stringent quality control measures were applied throughout the transcriptomic analysis, it should be noted that gene expression levels do not always directly reflect protein synthesis or activity. Future studies could integrate proteomic or immunological approaches to verify whether the observed transcriptional changes translate into altered enterotoxin production, particularly since gene expression may also vary with environmental factors such as temperature^46^. On the other hand, our study provides a comprehensive analysis of how different carbohydrates and incubation temperatures affect *B. cereus* ATCC 10987 strain growth and the regulation of key metabolic pathways, including the expression of enterotoxin genes.

## Conclusions

In this study, we confirmed the significant influence of temperature on *B. cereus* growth. Moreover, we demonstrated that lactose-rich environment not only slowed down the growth of *B. cereus* ATCC 10987 strain but also reduced the expression of key enterotoxin genes, namely *nhe* and *entFM* genes. In contrast, glucose, sucrose, and fructose supported robust growth, although glucose was associated with the downregulation of *nheB* and upregulation of *entFM*.

These findings are particularly relevant in the context of breastfeeding, suggesting that lactose present in human milk may play a protective role against alimentary *B. cereus s. l.* infections in infants. Future studies could explore the potential therapeutic or prophylactic applications of lactose in both pediatric and adult cases of *B. cereus*-related foodborne illness.

## Supplementary Information

Below is the link to the electronic supplementary material.

Supplementary Material 1

Supplementary Material 2

## Data Availability

The data for this study have been deposited in the European Nucleotide Archive (ENA) at EMBL-EBI under accession number PRJEB86181 (https://www.ebi.ac.uk/ena/browser/view/PRJEB86181)).
