# Supplementary material for: Effect of carbohydrate substrates on growth and enterotoxin gene expression in Bacillus cereus (pacificus)
Source: Sci Rep. 2025 Dec 10;16:1917. doi: 10.1038/s41598-025-31689-5 (PMC12804731; doi:10.1038/s41598-025-31689-5)
Supplement: Supplementary file 2 — Supplementary Material 2 [file 41598_2025_31689_MOESM2_ESM.docx]

# Supplement 1

# Effect of carbohydrate substrates on growth and enterotoxin gene expression in *Bacillus cereus* (*pacificus*)

Katerina Vyklicka^1^, Jiri Kucera^2^, Vojtech Barton^1^, Jan Bohm^1^, Petra Kubasova^1^, Kateřina Paskova^2^, Roman Remínek^3^, Zdenek Glatz^2^, Jan Lochman^2^, Filip Ruzicka^4^, Petra Borilova Linhartova^1*^

^1^RECETOX, Faculty of Science, Masaryk University, Kotlarska 2, Brno, Czech Republic

^2^Department of Biochemistry, Faculty of Science, Masaryk University, Kotlarska 2, Brno, Czech Republic

^3^Institute of Analytical Chemistry of the Czech Academy of Sciences, v. v. i., Veveri 97, Brno, Czech Republic

^4^Institute of Microbiology, St. Anne's University Hospital in Brno, Pekarska 53, Brno, Czech Republic

*Corresponding author

## **Table of Contents of Supplementary Information**

[Supplementary Methods 3](#_Toc199175204)

[Supplementary Figure 7](#_Toc199175207)

[Supplementary References 8](#_Toc199175208)

## Supplementary Methods

### Whole genome sequencing (WGS)

**DNA isolation from a single colony**

Genomic DNA was isolated from a single colony of *Bacillus cereus* ATCC 10987 grown on blood agar. Enzymatic lysis suitable for Gram-positive bacteria was combined with organic phase separation. The colony was resuspended in phosphate-buffered saline (PBS), centrifuged, and the resulting cell pellet was treated with lysis reagents: lysosyme (20 mg mL^-1^; Sigma-Aldrich), lysis solution (20 mM Tris–HCl, pH 8.0; 2 mM EDTA; 1.2% Triton X-100), and RNase (25 µL mL^-1^; Sigma-Aldrich). The suspension was incubated at 37 °C for 30 min. Subsequently, proteinase K (QIAGEN) and AL buffer (QIAGEN) were added, followed by incubation at 56 °C for 30 min.

Phase separation was initiated by two consecutive extractions with a phenol:chloroform:isoamyl alcohol (25:24:1, v/v/v). The aqueous phase was transferred to a new tube and extracted twice with chloroform:isoamyl alcohol (24:1, v/v). The resulting aqueous phase was again transferred to a fresh tube. Genomic DNA was precipitated with absolute ethanol, dried at 37 °C, and dissolved in TE buffer.

**DNA library preparation**

A genomic DNA library was prepared using the DNA Prep Tagmentation kit (Illumina) with Nextera DNA CD Indexes (Illumina), following the manufacturer’s protocol. Paired-end sequencing (2 × 300 bp) was performed using the MiSeq Reagent Kit v3 (600 cycles; Illumina) on the MiSeq platform (Illumina).

**Bioinformatic analysis**

WGS data were obtained in FASTQ format. Initial quality control of raw reads was performed using FastQC (v0.11.5) (Andrews, 2010), and results were summarized using MultiQC (v1.8) [2]. Key quality parameters, including per-base sequence quality, GC content, and sequence duplication levels, were assessed to ensure data reliability. Adaptor sequences and low-quality bases were trimmed using FastP (v0.23.2) [3], which also removed reads with a Phred quality score below 20. Only high-quality, paired-end reads were retained for downstream analysis.

High-quality reads were mapped to all complete genomes available in the NCBI Datasets under taxonomy ID 1396 (Supplementary Table S1) to confirm the identity of the *B. cereus* strain. Read alignment was performed using Bowtie2 (v2.5.1) [4]. The resulting BAM files were sorted and indexed using SAMtools (v1.19.1) [5].

For each mapping file, quality metrics were evaluated to determine the origin of the bacterial strain. The key parameters assessed included the number of mapped reads, the percentage of reads aligned to the reference genome, and the depth and uniformity of coverage. Strain identification was based on a comparative analysis of these mapping quality metrics across all reference genomes. The strain corresponding to the reference genome with the highest number of mapped reads and the most consistent coverage was selected as the best match.

The sequencing data generated in this study have been deposited in the European Nucleotide Archive (ENA) at EMBL-EBI under accession number PRJEB86181 (<https://www.ebi.ac.uk/ena/browser/view/PRJEB86181>). Raw FASTQ files are publicly available to ensure transparency and reproducibility of the results.

### Capillary electrophoresis (CE)

Samples stored at –80 °C were rapidly thawed in hot water and centrifuged at 12,000 × *g* for 10 min. Subsequently, 10 μL of the supernatant from each sample were diluted 10-fold with 5 g L^-1^ (19.2 mM) glucose-6-phosphate (Fluka) in deionized water. The selectivity of the method was confirmed by spiking the injected samples with corresponding analytical standards.

All analyses were performed using a 7100 CE system (Agilent Technologies) equipped with a photodiode array UV-Vis detector. The separations were accomplished in the 75 μm id fused‐silica capillary (113 cm total length, 104.5 cm effective length) thermostated at 37 °C. Before first use, the capillary was activated by rinsing with 1 M NaOH for 30 min followed by deionized water for 10 min at 50 °C. Prior to each analysis, the capillary was preconditioned by sequential rinsing with 0.1 M NaOH for 1 min, deionized water for 1 min, and background electrolyte (see below) for 3 min. After each run, the capillary was flushed with deionized water for 1 min. All rinsing procedures were carried out at a pressure of 950 mbar (95 kPa).

The sample solution was injected into the capillary at a pressure of 30 mbar (3 kPa) for three seconds. The plug volume was estimated using CE Expert Lite software (Beckman Coulter) to be 9 nL. Its length was calculated to be approximately 4.6 mm, representing about 0.4% of the total capillary length. Background electrolyte was composed of 0.27 mM CTAB prepared in 4.4 mM potassium hydrogen phthalate buffer with pH adjusted according to the monitored group of analytes: formate, acetate, propionate, and lactate – pH 5.5; glucose, fructose, galactose, lactose, sucrose, and butyric acid – pH 11.2; xylitol – pH 12.1 Separations were carried out under a voltage of -30 kV (–265.5 V cm^-1^, negative polarity) with a current of –8 μA. Analytes were detected indirectly at 200 nm with a reference wavelength set to 360 nm. Data acquisition and peak integration were performed using ChemStation software, version C.01.03 (Agilent Technologies).

### Statistical evaluation of results

The values presented in Table 1 (in the article) were determined from four independently prepared samples, each of which was analyzed in duplicate. In total, eight concentration values were obtained for each respective concentration of the carbon source or organic acid, respectively. The data were first tested for normality using the Shapiro-Wilk test and for homogeneity of variances using Levene's test. A one-way ANOVA was then performed, resulting in a p-value of 0 revealing that at least one group mean was significantly different from the others. To identify differences among the sample groups with different carbon sources, the Tukey's HSD test was applied. The test revealed statistically significant differences in carbon source consumption between the group of samples containing fructose, glucose, or sucrose, and those containing galactose, lactose, or xylitol (p-values ranged from 6.506×10^-12^ to 1.132×10^-8^). In case of organic acids production, a statistically significant difference between these groups was revealed for lactate (p-values ranges from 2.615×10^-13^ to 1.033×10^-10^), whereas for formate and acetate, significant differences were observed even among the carbon sources within the groups. All statistical tests were conducted at a significance level of α=0.05.

The values presented in Table 2 (in the main article) were determined as the mean of four measurements. Their statistical evaluation was carried out as described above, i.e., after the confirmation of data normality and homogeneity of variances, a one-way ANOVA and Tukey's HSD test were applied. As a result, statistically significant differences between groups of samples containing fructose, glucose, or sucrose, and those containing galactose, lactose, or xylitol, in the pH values after 12-hour incubation were revealed (p-values ranged from 2.599×10^-13^ to 1.013×10^-10^, α=0.05).

## Supplementary Figure


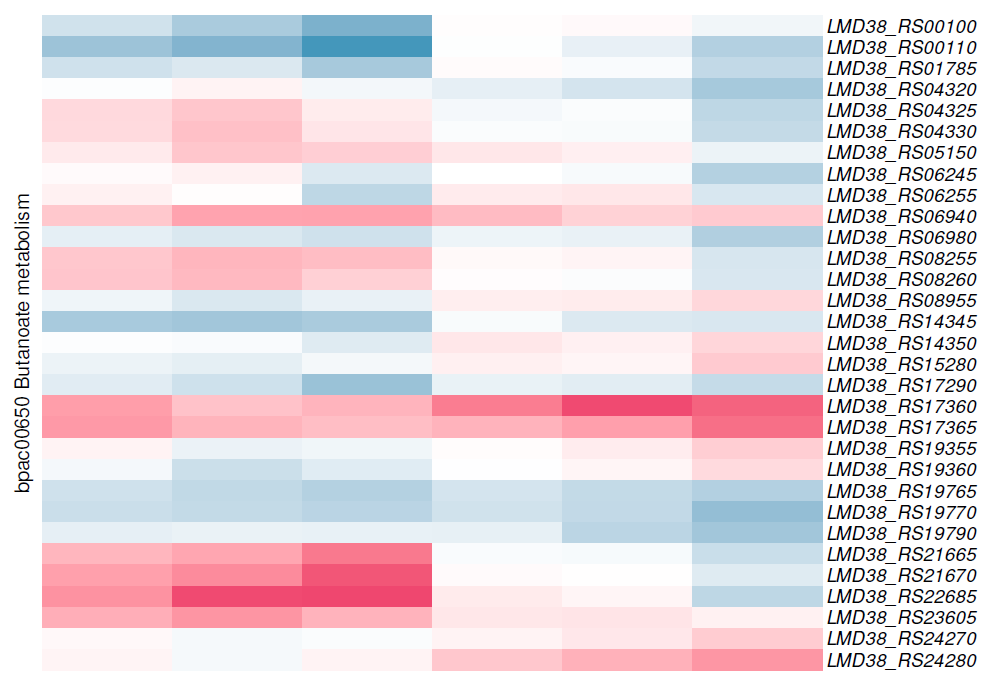

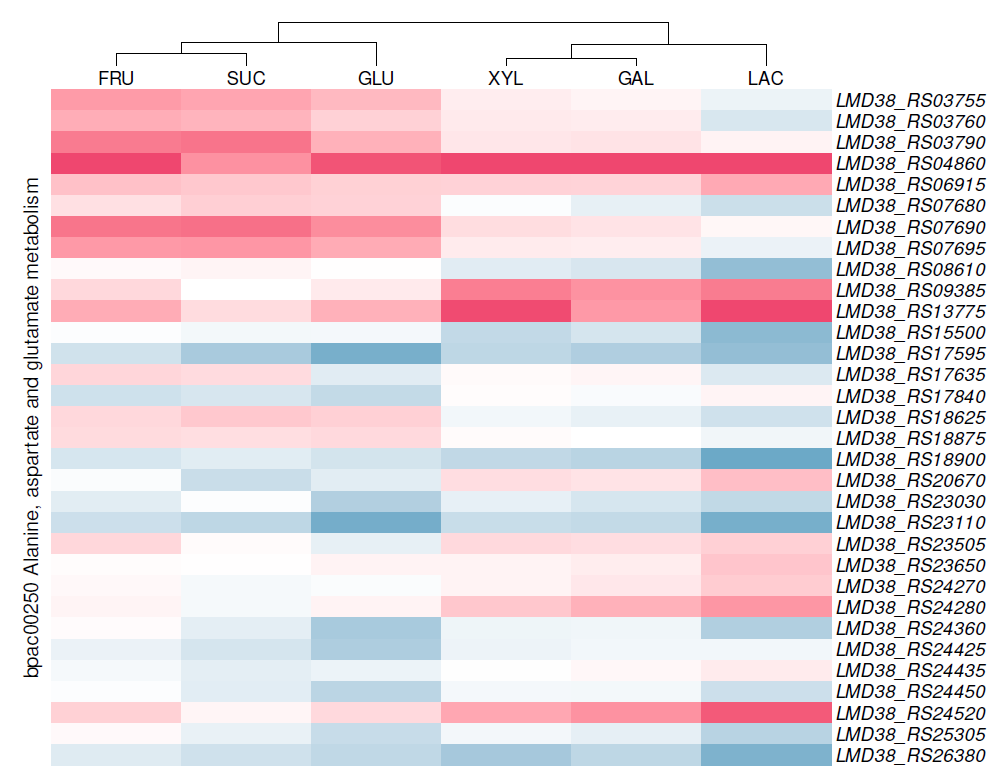

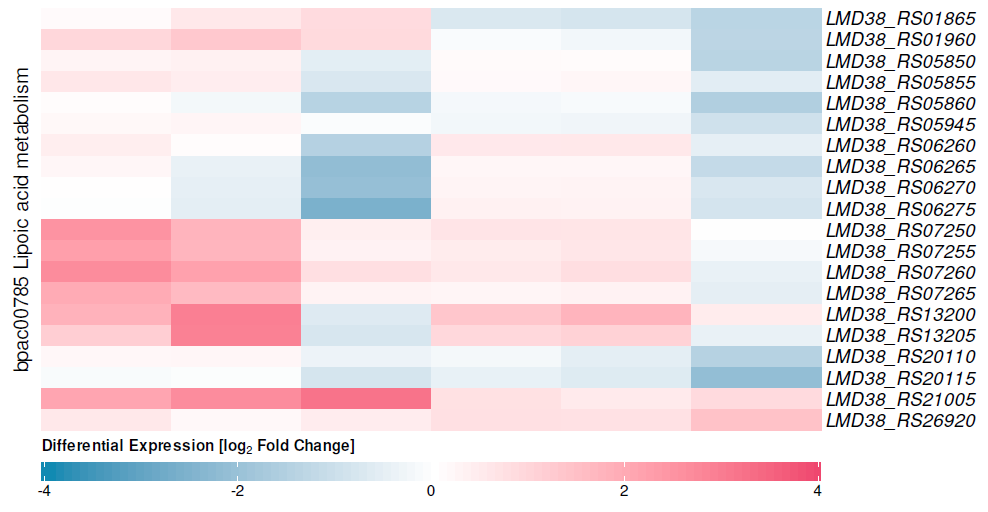


**Figure S1.** Heatmap of differentially expressed genes in *Bacillus cereus* (ATCC 10987) involved in three metabolic pathways across different carbohydrate substrates. FRU, fructose; GAL, galactose; GLU, glucose; LAC, lactose; SUC, sucrose; XYL, xylitol.

## Supplementary References

1. Babraham Bioinformatics. FastQC: A Quality Control tool for High Throughput Sequence Data [Internet]. [cited 2025 Feb 23]. Available from: https://www.bioinformatics.babraham.ac.uk/projects/fastqc/
2. Ewels P, Magnusson M, Lundin S, Käller M. MultiQC: summarize analysis results for multiple tools and samples in a single report. Bioinformatics [Internet]. 2016 [cited 2025 Feb 25];32(19):3047–8. Available from: https://doi.org/10.1093/bioinformatics/btw354
3. Chen S, Zhou Y, Chen Y, Gu J. fastp: an ultra-fast all-in-one FASTQ preprocessor. Bioinformatics [Internet]. 2018 [cited 2025 Feb 23];34(17):i884–90. Available from: https://doi.org/10.1093/bioinformatics/bty560
4. Langmead B, Salzberg SL. Fast gapped-read alignment with Bowtie 2. Nat Methods [Internet]. 2012 [cited 2025 Feb 23];9(4):357–9. Available from: https://www.nature.com/articles/nmeth.1923
5. Danecek P, Bonfield JK, Liddle J, Marshall J, Ohan V, Pollard MO, et al. Twelve years of SAMtools and BCFtools. GigaScience [Internet]. 2021 [cited 2025 Feb 25];10(2):giab008. Available from: https://doi.org/10.1093/gigascience/giab008
